# Public, animal, and environmental health implications of aquaculture.

**DOI:** 10.3201/eid0304.970406

**Published:** 1997

**Authors:** E S Garrett, C L dos Santos, M L Jahncke

**Affiliations:** National Seafood Inspection Laboratory, Pascagoula, Mississippi, USA.

## Abstract

Aquaculture is important to the United States and the world's fishery system. Both import and export markets for aquaculture products will expand and increase as research begins to remove physiologic and other animal husbandry barriers. Overfishing of wild stock will necessitate supplementation and replenishment through aquaculture. The aquaculture industry must have a better understanding of the impact of the "shrouded" public and animal health issues: technology ignorance, abuse, and neglect. Cross-pollination and cross-training of public health and aquaculture personnel in the effect of public health, animal health, and environmental health on aquaculture are also needed. Future aquaculture development programs require an integrated Gestalt public health approach to ensure that aquaculture does not cause unacceptable risks to public or environmental health and negate the potential economic and nutritional benefits of aquaculture.


### Public, Animal, and Environmental Health Implications of Aquaculture

**E. Spencer Garrett,* Carlos Lima dos Santos,† and Michael L. Jahncke‡**
*National Seafood Inspection Laboratory, Pascagoula, Mississippi, USA;
†Food and Agriculture Organization of the United Nations, Rome, Italy;
‡Virginia Tech Seafood Research and Extension Center,
Hampton, Virginia, USA

Aquaculture is important to the United States and the world's fishery system. Both import and export markets for aquaculture products will expand and increase as research begins to remove physiologic and other animal husbandry barriers. Overfishing of wild stock will necessitate supplementation and replenishment through aquaculture. The aquaculture industry must have a better understanding of the impact of the "shrouded" public and animal health issues: technology ignorance, abuse, and neglect. Cross-pollination and cross-training of public health and aquaculture personnel in the effect of public health, animal health, and environmental health on aquaculture are also needed. Future aquaculture development programs require an integrated Gestalt public health approach to ensure that aquaculture does not cause unacceptable risks to public or environmental health and negate the potential economic and nutritional benefits of aquaculture.

### U.S. Fisheries System

Coastal estuaries serve as a breeding ground and provide habitats for more than 75% of commercial landings and 80% to 90% of the recreational catch of fish and shellfish. From these habitats, hundreds of species of seafood are produced. Aquacultured species now contribute up to 15% of the U.S. supply (1,2). Wild species are harvested by 17,000,000 recreational anglers and nearly 300,000 commercial harvesters. Commercial harvesters deploy 93,000 vessels, while recreational fishermen have millions of recreational fishing boats. Nearly 5,000 domestic plants are located in every state throughout the United States, not just in the coastal areas (3). Current per capita consumption of commercially harvested species averages 15 pounds; it is estimated that per capita consumption of recreationally harvested seafood approaches an additional 3 to 4 pounds per person (4).

Address for correspondence: Michael Jahncke, Virginia Tech Seafood Research and Extension Center, 102 King Street, P.O. Box 369, Hampton, VA 23669 USA; fax: 757-727-4871; e-mail: mjahncke@vt.edu.

The seafood business community—in the United States and in other industrialized countries—cannot rely solely on domestically produced stock. For a number of years, more than half of U.S. seafood consumption has relied on imported stock. Currently, the United States imports more than 50% of the consumed seafood, which originates in 172 countries around the world (3). This trend toward economic reliance on imported stock has steadily increased over the past 10 years so that now the United States is the world's second largest importer of seafood. The principal seafood imports are tuna, shrimp, salmon, lobster, and groundfish (3).

It is difficult to determine where imported fish was harvested. For example, the United States imports salmon from Switzerland and Panama, although neither Switzerland nor Panama is noted for vast salmon resources. U.S. participation in the international seafood trade is very complex, since in addition to being the world's second largest importer, the United States is also the world's second largest exporter of seafood (3). This dichotomy requires that U.S. marketing and import/export food control inspection strategies be carefully planned. For example, the United States exports seafood to 162 countries, which has come about with the full development of northwest and Alaska fisheries and improved efficiency in processing techniques. Major U.S. exports are salmon, crab, surimi, fish blocks, groundfish, flatfish, shrimp, and lobster (3).

### Current Aquaculture Status

In 1996, U.S. aquaculture production of nearly 227,000 metric tons consisted of baitfish, catfish, salmon, trout, clams, crawfish, mussels, oysters, fresh and saltwater shrimp, and miscellaneous species such as ornamental fish, alligators, algae, aquatic plants, tilapia, and hybrid striped bass. The United States exported principally rainbow trout, Atlantic salmon, tilapia, catfish, freshwater crawfish, and live mussels to 19 countries in Europe, North and South America, and Asia. Freshwater crawfish led the export seafood market at slightly over $8 million, with the other species accounting for less than $1 million each (3). The United States also imports large volumes of aquacultured products, approximately $2.5 billion in cultured products, primarily shrimp and salmon. Imported cultured seafood accounts for most of the current U.S. trade deficit for edible fishery products, which was approximately $3.5 billion in 1995.

### The Safety of Seafood

Most seafood is safe; however, like all foods, it carries some risk. The food safety issues for seafood are highly focused, well-defined, and limited to a very few species. For seafood-borne illnesses (in which the cause was known) reported to the Centers for Disease Control and Prevention, more than 90% of the outbreaks and 75% of the individual cases were associated with ciguatoxin (from a few reef fish species) and scombrotoxin (from tuna, mackerel, bluefish, and a few other species) and the consumption of mollusks (mostly raw) (5-12).

Hazards associated with the consumption of all food (including seafood) can be categorized into three areas: product safety; food hygiene (clean vs. dirty plants, wholesome vs. unwholesome products); and mislabeling or economic fraud. Traditionally, the food safety risks of seafood products (aquacultured and wild-caught) have been subcategorized by environment, process, distribution, and consumer-induced risk; the environmental risk category is further subdivided into natural hazards (e.g., biotoxins) and anthropogenic contaminants (e.g., polychlorinated biphenyls) (13).

### “Shrouded” Aquaculture Hazards

The future of aquaculture is bright; aquaculture products are as safe and wholesome as wild-caught species. However, in addition to the consumer hazards listed above, there are some less obvious “shrouded” public health hazards associated with ignorance, abuse, and neglect of aquaculture technology.

### Technology Ignorance

A common practice in many developing countries is the creation of numerous small fish pond impoundments. However, this approach may have a greater adverse effect on human health than the creation of a single large impoundment (14). Small impoundments greatly increase the overall aggregate shoreline of ponds, causing higher densities of mosquito larvae and cercaria, which can increase the incidence and prevalence of diseases such as lymphatic filariasis and schistosomiasis, respectively. Centralized planning approaches for new freshwater and marine aquaculture sites should include discussions of the potential effect of large or small impoundments on such issues as disease transmission, water supply, irrigation, and power generation (14).

Ignorance of the microbial profile of aquaculture products can also affect human health as evidenced by the recent transmission of streptococcal infections from tilapia to humans, which resulted in several meningitis cases in Canadian fish processors (15). A change in marketing strategies to sell live fish in small containers, instead of ice-packs, resulted in human Vibrio infections from live tilapia in Israel in 1996. Such bacteria can be present in other aquacultured and wild-caught species in addition to tilapia.

Ignorance of the hazards associated with the use of untreated animal or human waste in aquaculture ponds to increase production also has tremendous human health implications (16). For centuries, food growers have cultured species in waste-water–fed ponds and grown secondary vegetable crops in waste water and sediment material in integrated aquaculture operations. However, the potential for transmission of human pathogens to cultured species and secondary vegetable crops is rarely considered by fishery aquaculturists. For example, of more than 250 presentations at the 1997 World Aquaculture Society meeting held in Seattle, Washington, few referred to the potential human health implications of aquaculture (17).

The potential transmission of animal pathogens from exotic aquacultured species to wild-stock species also affects animal health. Recent outbreaks of taura, yellow spot, and white head viruses have occurred in aquaculture shrimp in South Carolina and Texas. Recent studies indicate that native wild white shrimp may also be susceptible to these exotic viruses (18).

### Technology Abuse

Technology abuse includes the willful misuse of therapeutic drugs, chemicals, fertilizers, and natural fishery habitat areas. The widespread use and misuse of antibiotics to control diseases in aquaculture species is worldwide and will probably increase as aquaculturists move towards more intensive animal husbandry–rearing techniques and stocking densities. For example, the illegal use of chloramphenicol in shrimp culture to control diseases may result in violative levels in the harvested product. Similarly, the improper or illegal use of chemicals (e.g., tributyl tin) to control pond pests such as snails can also result in human health hazards. The abuse and misuse of raw chicken manure as pond fertilizer may result in the transmission of *Salmonella* from manure to the cultured product (16).

The destruction of mangrove areas to build aquaculture ponds can have a drastic impact on the survival of wild aquatic species through the degradation of essential fish habitats and nurseries. In Brazil, destruction of mangrove areas for shrimp ponds affected climatic changes to such an extent that the aquaculture operations have been terminated because consequent reduced rainfall resulted in excessive pond salinity (19).

### Technology Neglect

The final “shrouded” hazard associated with aquaculture involves technology neglect, which includes such events as the abandonment of small aquaculture ponds in tropical countries, leading to increased mosquito habitats and concomitant increases in malaria (14). Facility management can be responsible for technology neglect if employees are not trained in the proper use and application of therapeutics and chemicals, for example. Finally, from an animal health perspective, ignorance or willful neglect of the International Council for Explorations of the Sea/European Inland Fisheries Advisory Commission Code of Practice for the Introduction and Transfer of Marine Organisms can result in the escape of exotic species and animal pathogens into the environment with a potential tragic impact on native aquatic species (20).

### Health Control Considerations

### Human Health

Procedures to help protect humans from aquaculture-associated risks include better education and training of aquaculture personnel on the proper use and storage of therapeutics and chemical compounds. Additional research on new more effective and, we hope, safer, antibiotic and vaccine treatment of aquaculture species is under way. Likewise, certain extralabel use applications for selected antibiotics are under consideration. Streamlined enforcement efforts are being developed to ensure compliance with new food safety regulations and new regulatory control procedures such as Hazard Analysis and Critical Control Points (HACCP) and the application of HACCP principles to animal and environmental control procedures (21,22).

The Food and Agriculture Organization and World Health Organization recommend that the HACCP concept be applied to fresh water aquaculture programs to control foodborne digenetic trematode infections in humans. Experiments are being carried out in Asia by a multidisciplinary team of experts in public health, parasitology, aquaculture, fisheries extension, and fish inspection (22). In one study in Vietnam, experimental activities were conducted in two side-by-side fish ponds. In the experimental ponds, fish were cultured in conjunction with HACCP principles, and control pond fish were cultured according to conventional local aquaculture practices (22). Water supply, fish fry, fish feed, and pond conditions in the experimental pond were identified as critical control points. The HACCP principles of hazard analysis, preventative measures, critical limits, monitoring, recordkeeping, and verification procedures relating to the critical control points were applied; study results showed *Clonorchis*

*sinensis* eggs and fish infected with the parasite metacercaria and the first intermediate host (*Melanoides tuberculata*) in the experimental ponds (22). Forty-five percent of control pond fish were infected with *C. sinensis* metacercaria, while white fish from the experimental pond monitored according to HACCP principles were completely free of trematode infection (22). Preliminary results indicate that application of HACCP-based principles to silver carp culture in North Vietnam is an effective way to prevent and control *C. sinensis*. Similarly, the application of these principles to fresh water aquaculture ponds in Thailand and Laos to control *Opisthorchis viverrini* infections has also been successful. Additional studies are recommended to confirm these preliminary results (22).

### Animal Health

Procedures to safeguard animal health are set out in the International Council for Explorations of the Sea and the European Inland Fisheries Advisory Commission Codes of Practice, which describe how to prevent the adverse effects of introducing new and exotic species and emerging animal pathogens. Education and on-site training programs for aquaculture employees will help them understand the detrimental impact of introduction of exotic species and animal pathogens, misuse and abuse of therapeutics and chemicals, and willful habitat destruction. High priority issues also include implementation of biosecurity procedures in aquaculture operations to prevent the escape and spread of exotic species and pathogens into the facility and surrounding natural environment and the use of the HACCP principles to help control the spread of exotic pathogens to wild aquatic populations (17,23).

The application of HACCP principles to control transmission of exotic shrimp viruses from cultured to wild shrimp was proposed at a shrimp pathogen workshop held in June 1996 in New Orleans, Louisiana (21). Natural resource regulatory agencies are concerned about the possible transmission of exotic shrimp pathogenic viruses, recently found in shrimp aquaculture ponds in Texas and South Carolina, to wild native shrimp populations. The principles of HACCP, in conjunction with International Council for Explorations of the Sea and the European Inland Fisheries Advisory Commission Codes of Practice, were proposed to control the spread of exotic animal viruses into the environment. Shrimp aquaculture has the following proposed critical control points: pond site selection; water supply quality; pond management techniques; and transportation, especially as it relates to the live transport of aquaculture shrimp species (21).

Approximately 600 million pounds of shrimp are also imported for further processing into the United States on a yearly basis, half of which are aquacultured species (3). Natural resource managers, particularly at the state level, are concerned about the possible transmission of exotic shrimp pathogens into the environment from shrimp processing plant wastewater discharge and solid waste material landfill leakage. Proposed HACCP shrimp processing plant critical control points include unload/receive; de-ice/wash; thaw; dehead/peel/devein; wash; re-ice; de-ice/wash; re-ice; and dip/glaze (21).

Application of HACCP principles at aquaculture site and processing plant locations has the potential to control transmission of exotic human and animal pathogens. However, to our knowledge, except for the application of HACCP principles to control of human pathogens in Asia (17), no research has been conducted on this issue.

### References

1. Ratafia M. Aquaculture today: a worldwide status report. Aquaculture News 1994;3:12-13,18-19.
2. Rhodes RJ. Status of world aquaculture. Aquaculture Magazine 17th Annual Buyers Guide 1987;4-18.
3. National Marine Fisheries Service. Fisheries of the United States 1995. Current Fisheries Statistics No. 9500. Washington (DC): U.S. Department of Commerce; 1996.
4. Krebs-Smith SM. Report on per capita consumption and intake of fishery products [letter]. In: National Academy of Sciences Committee on the Evaluation of the Safety of Fishery Products 1991 Seafood Safety Report. Washington (DC): National Academy Press; 1991.
5. Centers for Disease Control. Annual summary of foodborne diseases (1978). Atlanta (GA):CDC;1979.
6. Centers for Disease Control. Annual summary of foodborne diseases (1979). Atlanta (GA):CDC;1981.
7. Centers for Disease Control. Annual summary of foodborne diseases (1981). Atlanta (GA):CDC;1983.
8. Centers for Disease Control. Annual summary of foodborne diseases (1980 and 1981). Atlanta (GA):CDC;1983.
9. Centers for Disease Control. Annual summary of foodborne diseases (1982). Atlanta (GA):CDC;1985.
10. Centers for Disease Control. Annual summary of foodborne diseases, unpublished data. Atlanta (GA):CDC;1989.
11. Centers for Disease Control. Foodborne disease outbreaks, 1983-87. MMWR CDC Surveill Summ 1990;39(SS-1):1-45.

12. National Marine Fisheries Service. The draft report of the model seafood surveillance project. A report to Congress. Pascagoula (MS): Office of Trade and Industry Services, National Seafood Inspection Laboratory;1995.
13. Garrett ES. Role of diseases in marine fisheries management. Transactions of the 50th North American Wildlife and Natural Resources. NOAA Conference 1986. Tech. Memo. NMFS, F/NWR-16. Washington (DC): National Marine Fisheries Service;1986.
14. Mott KE. A proposed national plan of action for schistosomiasis control in the United Republic of Cameroon. Geneva: World Health Organization (unpublished document WHO/SCHISTO/86,88); 1996.
15. Centers for Disease Control and Prevention. Invasive infection with *Streptococcus iniae*-Ontario, 1995-1996. MMWR Morb Mort Wkly Rep 1996; 45(30):650-3.
16. Buras N. Microbial safety of produce from wastewater fed aquaculture. In: Pullin RVC, Rosenthal H, Maclean JL, editors. Environment and aquaculture in developing countries. Proceedings of the 31st International Center for Living Aquatic Resources Management Conference; 1993; Manila, Philippines. Manila: The Center; 1993. p. 285-95.
17. World Aquaculture Society. Abstracts of the Annual International Conference and Exposition of the World Aquaculture Society and The National Aquaculture Conference and Exposition of National Aquaculture Association; 1997 Feb 19-23; Seattle, WA.
18. Virus re-emerges at shrimp farm. The Aquaculture News 1996;4(11):1-15.
19. Pullin RSV. Discussion and recommendations on aquaculture and the environment in developing countries. In: Pullin RSV, Rosenthal H, Maclean JL, editors. Environment and aquaculture in developing countries. Proceedings of the 31st ICLARM Conference 1993. p. 312-338.
20. Turner GE, editor. Codes of practice and manual of procedures for consideration of introductions and transfers of marine and freshwater organisms. EIFAC Occasional Paper 23. Rome: FAO; 1988.
21. Jahncke JL. The application of the HACCP concept to control exotic shrimp viruses. Proceedings of the NMFS Workshop on Exotic Shrimp Viruses. 1996 June; New Orleans, LA.
22. Son TQ, Hoi VS, Dan TV, Nga C, Toan TQ, Chau LV, et al. Application of hazard analysis critical control point (HACCP) as a possible control measure against *Clonorchis sinensis* in cultured silver carp *Hypophthalmichthys molitrix.* Paper presented at 2nd Seminar on Food-Borne Zoonoses: Current Problems, Epidemiology and Food Safety; 1995 Dec 6-9; Khon Kaen, Thailand.
23. Austin B. Environmental issues in the control of bacterial diseases of farmed fish. In: Pullin RVC, Rosenthal H, Maclean JL, editors. Environmental and aquaculture in developing countries. Proceedings of the 31st International Center for Living Aquatic Resources Management Conference; 1993; Manila, Philippines. Manila: The Center; 1993. p. 237-51.
